# Characterisation and outcomes of patients referred to a regional cancer of unknown primary team: a 10-year analysis

**DOI:** 10.1038/s41416-021-01544-1

**Published:** 2021-09-06

**Authors:** Mark Stares, Karin Purshouse, Gillian Knowles, Rachel Haigh, Jenny Irvine, Aoife Gatenby, Rebekah Patton, Jo McGinty, Alan Christie, Marjory MacLennan, Colin Barrie, Sally Clive

**Affiliations:** 1 Edinburgh Cancer Centre, Western General Hospital, NHS Lothian, Edinburgh, UK; 2 University of Edinburgh, Edinburgh, UK

**Keywords:** Cancer of unknown primary, Cancer of unknown primary

## Abstract

**Background:**

In the United Kingdom, national guidance published in 2010 recommended the establishment of specialist teams to improve clinical pathways for patients presenting with malignancies of undefined primary origin (MUO) and cancer of unknown primary (CUP). This study sought to define outcomes of patients referred to a regional MUO/CUP service.

**Methods:**

Data were collected prospectively on all patients (*n* = 1225) referred to a regional CUP team over a 10-year period. Patient demographics, clinical, pathological and outcome data were recorded and analysed.

**Results:**

Confirmed CUP (cCUP) was diagnosed in 25% of patients. A primary metastatic cancer was identified in 36%, 5% were diagnosed with provisional CUP (pCUP), 27% retained the diagnosis of MUO and in 8% a non-cancer diagnosis was made. Median survival was low in all patients with a final malignant diagnosis: primary identified 9.0 months, cCUP 4.0 months, pCUP 1.5 months and MUO 1.5 months.

**Conclusions:**

Patients presenting with MUO have poor outcomes irrespective of the final diagnosis. These patients need a patient-centred, streamlined, rapid diagnostic pathway. There are clear benefits to primary and secondary care teams having access to a dedicated, multidisciplinary MUO/CUP service, with clinical nurse specialists supporting the patients, to help facilitate this pathway and ensure early oncology review.

## Background

Patients presenting with a metastatic malignancy identified on clinical or radiological examination without an obvious primary site are said to have a malignancy of undefined primary origin (MUO) [1–3]. These patients may present to healthcare professionals in any clinical speciality, with over half diagnosed during emergency admissions to acute healthcare services [1]. Unlike patients with an obvious primary, in whom there are established multidisciplinary teams (MDTs), clear investigative pathways and monitored cancer targets, patients presenting with MUO can have protracted pathways to diagnosis and management, long hospital stays and be discussed by multiple MDTs. Whilst many of these patients will be found to have a defined underlying primary cancer after further investigations, some will not and will end up with a diagnosis of cancer of unknown primary (CUP). Historically, oncology teams have seen patients to discuss treatments once a confirmed histological diagnosis has been made, and after discussion at a cancer MDT meeting.

In the United Kingdom (UK) the National Institute for Clinical Excellence (NICE) published guidelines in 2010 to subclassify patients with MUO and assist healthcare professionals in their management [2]. Patients who have never had a biopsy, often because they are too unwell or comorbid, retain the diagnosis of MUO. Patients who undergo further investigations, including biopsy, will be diagnosed with either: a defined primary cancer, a non-cancer diagnosis or a diagnosis of cancer of unknown primary [2, 3, 4]. Patients with CUP have pathological evidence of malignancy but no confirmation of a primary cancer site, despite a standardised comprehensive diagnostic workup in accordance with published guidelines [2, 3]. CUPs may be defined further into provisional CUP (pCUP) or confirmed CUP (cCUP) dependent on the extent of investigation and whether the patient has been reviewed by an oncologist with a specialist interest in CUP [2]. Whilst this terminology is well recognised by those working in acute oncology and CUP teams, it is less well known by non-specialist teams, and the diagnosis of ‘CUP’ can be used somewhat indiscriminately. CUP represents ~2% of all new cancer diagnoses in the UK, and despite being only the 15th most common cancer by incidence, it is the 6th most common cause of cancer death [5].

The 2010 NICE guidelines for MUO/CUP recommended that every hospital with a Cancer Centre should establish a CUP team [2]. As a minimum, this should consist of an oncologist, a palliative care physician and a CUP specialist nurse or key worker. The key roles of the CUP team are to provide advice on appropriate investigations, multidisciplinary review of cases and help reach a working diagnosis from which to coordinate anti-cancer-specific treatments or recommend best supportive care.

Patients in whom a primary cancer is subsequently identified may be referred to site-specific MDTs for further management. In those diagnosed with CUP, ~20% fall into the ‘favourable prognosis’ group (i.e. single resectable metastatic site, extragonadal germ cell syndrome, neuroendocrine cancers, squamous neck or inguinal lymph nodes, axillary node adenocarcinoma in females, adenocarcinoma with colorectal phenotype, peritoneal disease in females with serous or papillary histology, bone metastases with high prostate-specific antigen (PSA) in males), which share clinicopathological characteristics with particular known metastatic cancers [3, 6, 7]. Median survival in this group may be as long as 24 months with treatment directed at the likely primary site, usually under the supervision of the relevant site-specific MDT [8–10]. In the remaining 80%, classed as ‘poor prognosis CUP’, presenting with adenocarcinoma and poorly differentiated carcinomas, treatment decisions are usually made by the supervising CUP team. Response to systemic therapy is often limited, with a median survival of 6–9 months in patients fit enough to attend specialist CUP clinics and to be recruited to clinical trials, but as low as 1–3 months in those presenting as emergencies [6, 11–13].

Despite the development of acute oncology teams and formal MUO/CUP services throughout the UK over the past decade, reliable outcome data for patients referred to these teams is lacking. A greater understanding of this patient group will help plan services and inform healthcare professionals when making decisions about investigations and management. The Edinburgh Cancer Centre (ECC) has an established MUO/CUP service with a dedicated MDT. It accepts primary and secondary care referrals from across the South East of Scotland, covering a total population of ~1.5 million. Referral criteria include a radiological investigation suspicious for cancer with no primary site easily identifiable and no recent cancer history. All referred patients are discussed at the regional MUO/CUP MDT and the team helps expedite appropriate investigations and biopsies where indicated. The ECC CUP team has routinely collected clinical information about patients referred to the service since its inception in 2010, as part of the ECC CUP Bio Study. We performed a 10-year analysis of the clinical and demographic data of this cohort to define the MUO/CUP population, understand their clinical pathways and assess survival in recognised subgroups in a real-world setting.

## Methods

### Study population

Prospective data collection was undertaken of all patients referred to the ECC CUP service between 1 September 2010 and 31 August 2020. Eligible patients were 18 years or over and had initially presented with MUO, defined as radiological suspicion of metastatic malignancy without an obvious primary site or recent cancer diagnosis. All patient cases were discussed in the ECC CUP MDT, with central pathology and radiology review, and almost all patients received support from MUO/CUP clinical nurse specialists (CNSs).

Patients were divided into final diagnosis subgroups for further analysis based on NICE guidelines [2]. Investigations to identify a primary site included as a minimum: comprehensive history and physical examination, blood tests, radiological imaging and histological examination of tumour tissue/cells (or elevated PSA in males with presentations compatible with prostate cancer), as guided by the clinical picture. Further specific investigations such as endoscopy, mammography, cancer markers or positron emission tomography scans were performed in accordance with published guidelines [2, 3]. Patients who did not undergo biopsy or comprehensive investigation (due to frailty, clinical deterioration or patient wishes) retained the diagnosis of MUO. Comprehensive investigations led to a diagnosis of either ‘non-cancer’, ‘primary cancer identified’, ‘pCUP’ or ‘cCUP’. ‘Non-cancer’ was given to those in whom biopsy confirmed an alternative diagnosis to cancer and/or a non-malignant diagnosis was established after independent clinical and radiology review within the ECC CUP MDT. ‘Primary cancer identified’ was given to those with a clinical, radiological and histological/biochemical pattern of a specific primary cancer as determined by the ECC CUP MDT and confirmed by the primary site-specific MDT. ‘pCUP’ was given to those with histological evidence of malignancy but no primary site identifiable after comprehensive investigation as determined by the ECC CUP MDT, but who were not fit enough to attend for further specialist review. ‘cCUP’ was given to those meeting the criteria for pCUP who also attended the ECC CUP clinic for review and further management by a consultant oncologist with a specialist interest in MUO/CUP.

### Procedure and assessment

Patient demographics, clinical, radiological and pathological data were recorded. All data were collected as part of routine oncology workup in keeping with standard care. No patient identifiable data were used. The presented work was in accordance with guidelines from ACCORD (Academic and Clinical Central Office for Research and Development, NHS Lothian and the University of Edinburgh) and ECC CUP-specific consent was not required. As the study was not designed to test a formal hypothesis, a sample size calculation was not required; all patients referred during the time period were assessed, irrespective of the final diagnosis.

### Statistical analysis

The overall survival, defined as the number of months from the first radiological evidence of MUO until death, or censorship if alive at follow-up date, was calculated.

Survival curves were plotted using Kaplan–Meier methods and log-rank tests applied. Statistical analyses were performed using IBM SPSS Statistics version 25 and GraphPad Prism version 8.4.2.

## Results

Clinical characteristics at the time of referral of 1225 patients referred to the ECC CUP team over the 10-year period are presented in Table 1. Fifty-two per cent were female and the median age was 72 years (interquartile range (IQR) 62–79 years).

**Table 1:** Clinical characteristics of patients referred to the Edinburgh Cancer Centre Cancer of Unknown Primary team over 10 years.

	All	Non-cancer diagnosis	Primary cancer identified	Confirmed CUP	Provisional CUP	MUO
	*n* (%)	*n* (%)	*n* (%)	*n* (%)	*n* (%)	*n* (%)
Patients	1225	97 (8)	443 (36)	301 (25)	56 (5)	328 (27)
*Demographics*						
Age						
≤65	399 (33)	41 (42)	193 (44)	124 (41)	10 (8)	31 (9)
66–74	329 (27)	19 (20)	121 (27)	94 (31)	18 (32)	77 (23)
>74	497 (41)	37 (38)	129 (29)	83 (28)	28 (50)	220 (67)
Median (IQR)	72 (62–79)	71 (58–79)	68 (58–76)	68 (60–75)	76 (70–83)	79 (72–84)
Sex						
Female	637 (52)	56 (57)	236 (53)	155 (51)	36 (64)	154 (47)
Male	588 (48)	41 (42)	207 (47)	146 (49)	20 (36)	174 (53)
*Referral pathway*						
Referral route						
Inpatient	740 (60)	45 (46)	239 (54)	163 (54)	44 (79)	249 (76)
Outpatient	485 (40)	52 (54)	204 (46)	138 (46)	12 (21)	79 (24)
*Presenting symptoms*						
Symptoms prompting investigations						
Symptomatic	1151 (94)	79 (81)	416 (94)	289 (96)	54 (96)	313 (95)
Incidental	74 (6)	18 (19)	27 (6)	12 (4)	2 (3)	15 (5)
*Initial radiological findings*						
Number of metastatic sites						
1	469 (38)	70 (72)	169 (38)	100 (33)	26 (46)	104 (32)
2	329 (27)	14 (15)	115 (26)	94 (31)	10 (18)	96 (29)
3	245 (20)	11 (11)	95 (21)	60 (20)	13 (23)	66 (20)
4+	182 (15)	2 (2)	64 (14)	47 (16)	7 (13)	62 (19)
Number of lesions						
1 (single lesion)	91 (7)	22 (26)	32 (7)	14 (5)	1 (2)	21 (6)
Sites of disease						
Visceral/bone only	705 (58)	78 (80)	234 (53)	150 (50)	32 (57)	211 (64)
Lymph node only	84 (7)	8 (8)	39 (9)	28 (9)	5 (9)	4 (1)
Visceral/bone and lymph node	436 (36)	11 (11)	170 (38)	123 (41)	19 (34)	113 (34)
*Survival*						
Survival (months)						
Median (IQR)	4.3 (1.6–16.0)	Not reached	9.0 (3.3-35.5)	4.0 (2.0–10.0)	1.5 (1.1–3.4)	1.5 (0.8–3.4)
Alive at 1 month	1058 (86)	97 (100)	418 (94)	280 (93)	44 (79)	219 (67)
Alive at 3 months	732 (60)	93 (96)	340 (77)	187 (62)	16 (29)	96 (29)
Alive at 12 months	349 (29)	82 (85)	182 (41)	63 (21)	2 (4)	20 (6)

A primary metastatic cancer was identified in 443 (36%) of patients, a non-cancer diagnosis was made in 97 (8%) and cCUP was ultimately diagnosed in 301 (25% of patients). Three hundred and twenty-eight (27%) patients did not undergo comprehensive investigation, and thus had a final diagnosis of MUO (Supplementary Figure 1). The most frequent reason for this was ‘frailty’ (*n* = 262, 80%), broadly defined as poor performance status (PS) and/or clinical unsuitability for invasive investigation or SACT (Supplementary Table 1). A further 56 (5%) patients underwent comprehensive investigation, but were not reviewed by a CUP oncologist (usually because of rapid clinical decline), giving them a final diagnosis of pCUP. Patients with pCUP or MUO were older than those with other diagnoses (median 79 (IQR 72–84) vs 68 (IQR 58–76) years; *p* < 0.001).

Sixty per cent of patients were referred during inpatient admissions following emergency presentations. Forty-five per cent of all referrals came from inpatient medical speciality healthcare teams (Supplementary Table 2). Patients with pCUP or MUO were more frequently referred during emergency inpatient admissions than patients with other final diagnoses (76 vs 53%) (*p* < 0.001). Symptoms prompted initial investigations in the majority of patients (97%), with pain (53%) and weight loss (42%) frequently described (Supplementary Table 3). One hundred and twenty-four (10%) patients presented with oncological emergencies, including spinal cord compression (*n* = 55) and/or hypercalcaemia of malignancy (*n* = 51). Outpatient referrals included 132 (27%) direct referrals from General Practitioner to the ECC CUP team and 112 (23%) referrals from other cancer MDTs.

The majority (*n* = 756 (62%)) of patients had more than one site of disease, with visceral organ or bone lesions seen in 1141 (93%). Liver lesions were the most frequent site of disease, seen in 501 (41%) patients including 192 (59%) of those with a final diagnosis of MUO (Supplementary Table 4). In almost all cases (*n* = 491 (98%)) multiple liver lesions were present. Bone was the most frequent single site of disease (*n* = 126 (27%)). Thirty-three (32%) solitary lesions were bone lesions, with 13 (43%) of these ultimately found to be non-malignant.

**Fig. 1:**
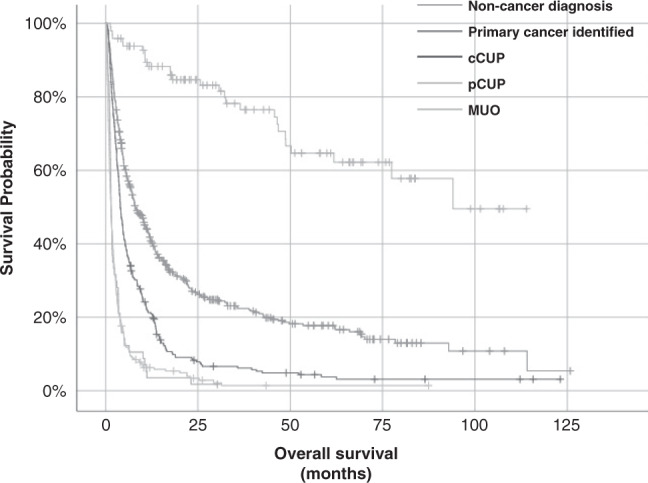
Kaplan–Meier curves examining survival for all diagnostic groups. *p* < 0.001 (log rank).

Survival was poor amongst all patients with a final diagnosis of confirmed or suspected malignancy (Fig. 1). At the time of censoring, 192 (16%) of patients were alive. The minimum and the median follow-up for survivors were 8.3 and 34.2 months, respectively. Median survival was not reached in the non-cancer diagnosis group despite a median follow-up time of 34.8 months. The median survival of patients with MUO was 1.5 (IQR 0.8–3.4) months and pCUP was 1.5 (IQR 1.1–3.4) months. Forty-six per cent (*n* = 26) of patients with pCUP died within 30 days of diagnostic biopsy. One-quarter of patients in our cohort had a final diagnosis of cCUP. The median survival of these patients was 4.0 (IQR 2.0–10.0) months, although notably, survival exceeded 12 months in 63 (21%) patients. Across all patient groups with a confirmed or suspected malignant final diagnosis, median survival for those referred during inpatient admissions was less than half that of patients referred via an outpatient route (*p* < 0.05) (Table 2 and Supplementary Figure 2).

**Table 2:** The relationship between the route of referral and survival in each final diagnostic subgroup.

	Survival (months)	*p* value	Alive at 3 months	Alive at 12 months
	Median (IQR)		*n* (%)	*n* (%)
All (*n* = 1225)				
Inpatient referral	2.8 (1.2–8.1)	<0.001	353 (48)	132 (18)
Outpatient referral	10.0 (3.5–35.7)		379 (78)	215 (44)
Non-cancer diagnosis (*n* = 97)				
Inpatient referral	Not reached	N/A	43 (96)	33 (73)
Outpatient referral	Not reached		50 (96)	47 (91)
Primary cancer identified (*n* = 443)				
Inpatient referral	5.6 (2.3–23.4)	<0.001	161 (67)	75 (31)
Outpatient referral	13.2 (5.4–49.7)		179 (88)	107 (52)
Confirmed CUP (*n* = 301)				
Inpatient referral	3.1 (1.6–5.4)	<0.001	83 (51)	15 (9)
Outpatient referral	6.8 (3.0–13.7)		104 (75)	48 (35)
Provisional CUP (*n* = 56)				
Inpatient referral	1.5 (1.0–2.4)	0.015	10 (23)	0 (0)
Outpatient referral	3.1 (1.3–10.6)		6 (50)	2 (17)
MUO (*n* = 328)				
Inpatient referral	1.3 (0.7–2.7)	<0.001	56 (22)	9 (4)
Outpatient referral	3.1 (1.5–6.3)		40 (65)	11 (14)

*N/A* not available.

For patients in whom a primary cancer was identified, the range of confirmed primary sites was broad (Table 3). Hepatobiliary/pancreatic (HPB) cancers comprised the largest single group, accounting for 58 (13%) cases. A significant proportion of patients (*n* = 72 (16%)) were found to have a primary haematological malignancy (i.e. lymphoma or myeloma). The median survival of all patients in whom a primary cancer was identified was 9.0 (IQR 3.3–33.5) months. However, when non-epithelial malignancies (e.g. lymphoma, myeloma, sarcoma, melanoma) were excluded median survival fell to 7.7 (IQR 3.2–21.9) months. Indeed, patients in whom a non-epithelial primary malignancy was identified represented a group presenting as MUO who have a more favourable survival of 32.9 (IQR 3.9–92.9) months (Supplementary Figure 3).

**Table 3:** Primary site in patients in whom a primary cancer was identified, histological subgroups of cCUP and associated median survival.

	*n* (%)	Survival (months) median (IQR)
Primary cancer identified—confirmed primary site		
Lung	58 (13)	5.6 (2.6–12.1)
Lymphoma	57 (13)	69.3 (5.8–78.4)
Hepatobiliary or pancreatic	54 (12)	4.3 (2.3–8.7)
Lower gastrointestinal	42 (9)	10.0 (4.3–22.0)
Urological	40 (9)	11.9 (5.0–39.4)
Upper gastrointestinal	34 (8)	3.2 (1.9–5.4)
Breast	31 (7)	15.1 (5.6–N/A)
Gynaecological	22 (5)	13.3 (4.5–40.2)
Sarcoma	20 (5)	30.5 (6.0–53.5)
Melanoma	17 (4)	3.5 (2.0–11.6)
Brain	15 (3)	10.3 (4.3–16.0)
Myeloma	15 (3)	92.9 (16.3–114.2)
Neuroendocrine tumour	13 (3)	21.9 (4.4–N/A)
Thyroid	5 (10)	26.7 (26.7–46.7)
Mesothelioma	2 (0)	16.6 (16.6–23.2)
Other	18 (4)	24.1 (4.4–N/A)
cCUP—most likely primary sites based on morphology and immunohistochemistry		
Upper gastrointestinal/hepatobiliary or pancreatic	101 (34)	3.1 (1.4–6.8)
Poorly differentiated/undifferentiated	65 (22)	4.0 (1.6–7.6)
Lower gastrointestinal	32 (11)	3.8 (2.2–11.3)
Lung/hepatobiliary or pancreatic	24 (8)	3.4 (2.4–11.2)
High-grade neuroendocrine tumour	21 (7)	3.9 (1.7–9.5)
Lung/upper gastrointestinal/hepatobiliary or pancreatic	17 (6)	3.5 (1.7–6.7)
Squamous cell carcinoma	17 (6)	3.4 (1.9–4.8)
Gynaecological	7 (2)	10.1 (2.6–18.2)
Breast/urothelial	3 (1)	3.7 (1.7–4.0)
Small cell carcinoma	3 (1)	10.0 (9.8–39.5)
Other	11 (4)	2.1 (6.7–12.4)
cCUP clinicopathological subgroup		
Favourable prognosis cCUP	74 (25)	5.5 (3.2–16.4)
Poor prognosis cCUP	227 (75)	3.7 (1.8–8.5)

*N/A* not available.

Comprehensive investigation, including extensive immunohistochemistry (IHC) analyses, gave a ‘best-fit’ tissue of origin in patients with cCUP (Table 3). Almost half of all cases (*n* = 142 (47%)) were found to have features that could be consistent with HPB cancer. Poorly differentiated or undifferentiated tumours accounted for a further 65 (22%) cases. Seventy-four (25%) cases had features of favourable prognosis cCUP [3]. Median survival of patients with favourable prognosis cCUP was 5.5 (IQR 3.2–16.4) months compared to only 3.7 (IQR 1.8–8.5) months in patients with poor prognosis cCUP (*p* < 0.001) (Supplementary Figure 4).

Two hundred and thirty-four (53%) patients in whom a primary was identified went on to receive systemic anti-cancer therapy (SACT). Only 86 (29%) patients with cCUP received SACT, including 32 (43%) with favourable cCUP and 54 (24%) with poor prognosis cCUP. Survival was improved in all patient groups who received SACT (*p* < 0.05) (Table 4). There was, however, no difference in survival between patients with favourable prognosis cCUP who received SACT and patients with poor prognosis cCUP who received SACT (12.6 (IQR 5.3–16.6) vs 10.1 (IQR 5.8–14.9), respectively (*p* = 0.101)) (Supplementary Figure 5). Three hundred and fourteen (26%) patients received palliative radiotherapy, including 167 (38%) of those in whom a primary cancer was diagnosed, 98 (33%) of those with cCUP and 49 (15%) of those with a final diagnosis of MUO.

**Table 4:** The relationship between treatment with SACT in patients with a final diagnosis of ‘primary cancer identified’ or ‘cCUP’.

	Patients	Survival (months)	*p* value	Alive at 3 months	Alive at 12 months
	*n* (%)	Median (IQR)		*n* (%)	*n* (%)
Primary cancer identified					
SACT	234 (53)	16.2 (7.7–68.8)	<0.001	222 (95)	137 (59)
No SACT	209 (47)	3.7 (1.8–10.8)		118 (56)	45 (22)
All cCUP					
SACT	86 (29)	10.9 (5.8–15.8)	<0.001	80 (93)	41 (48)
No SACT	215 (71)	3.0 (1.6–5.3)		107 (50)	22 (10)
Favourable prognosis cCUP					
SACT	32 (43)	12.6 (5.3–16.6)	0.040	29 (91)	17 (53)
No SACT	42 (57)	3.9 (2.2–12.4)		28 (67)	10 (24)
Poor prognosis cCUP					
SACT	54 (24)	10.1 (5.8–14.9)	<0.001	51 (94)	24 (44)
No SACT	173 (76)	2.8 (1.6–4.7)		79 (46)	12 (7)

*SACT* systemic anti-cancer therapy.

**Fig. 2:**
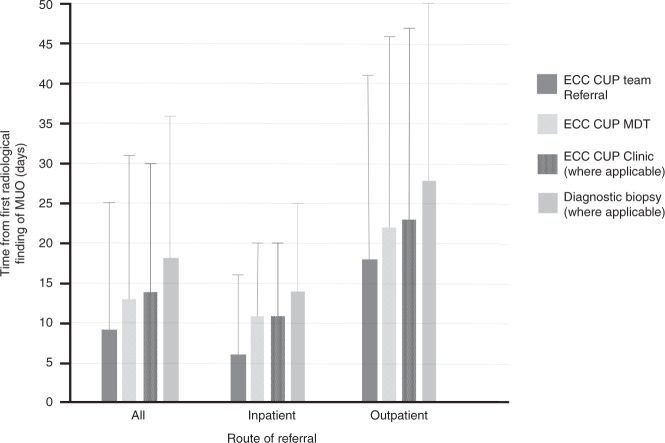
Time (days) from first radiological suspicion of MUO to key timepoints in the patient journey. Error bars represent interquartile range.

The median time between the first radiological suspicion of MUO and referral to the ECC CUP team was 9 (IQR 3–25) days (Fig. 2). However, the time to referral to the CUP team was three times longer for outpatient referrals compared to inpatient referrals (18 (IQR 5–41) vs 6 (IQR 2–16) days, respectively (*p* < 0.001). Following referral to the ECC CUP team subsequent time to first ECC CUP MDT (median 4 days), first attendance to ECC CUP clinic (where applicable) (median 5 days) and first diagnostic biopsy (where applicable) (median 9 days) were similar for patients referred as outpatients or inpatients.

## Discussion

We present the largest prospectively recorded and evaluated cohort of patients referred to an MUO/CUP service in the UK. Our data define the outcomes of these patients, who may be encountered by healthcare professionals across a broad range of medical and surgical specialities. These patients present with a diverse range of symptoms, clinical and radiological findings. Their poor outcomes reflect aggressive cancer behaviour and late diagnosis and highlight the need for rapid and realistic diagnostic pathways, coordinated care and early CNS support.

In patients presenting with radiological suspicion of metastatic disease with no obvious primary site, an initial assessment of patient fitness and wishes will guide ongoing management. When clinically appropriate, investigations should be carried out in an efficient manner with the aim of reaching a diagnosis or ‘best-fit’ pathological subtype as soon as possible, to inform prognosis and direct treatment [2]. However, the avoidance of over-investigation in patients with the most limited prognoses is equally important to enable informed discussions with the patient about the realities of advanced cancer and the realistic option of prioritising symptom management, palliative care and end of life planning over invasive investigations. In our cohort, approximately one in four patients did not undergo comprehensive investigations, with the majority considered clinically unsuitable for such procedures or for anti-cancer therapies. This patient group had the poorest prognosis (median 1.5 months), suggesting that potentially invasive investigations or hospital stays were rationally avoided. However, a small number of patients received palliative radiotherapy, highlighting the role of active symptom control strategies and involvement of specialist oncology services as part of their management.

In those patients who did have a biopsy but were then not fit enough to attend the CUP clinic (final diagnosis of pCUP), half died within 30 days of their diagnostic biopsy and survival matched those with MUO, at only 1.5 months. The majority of these patients were investigated during inpatient stays, but were not referred to the CUP team until after biopsy, suggesting an ongoing desire to ‘hunt the primary’ or reach a pathological diagnosis despite clinical frailty and the apparent futility of this approach. These inpatients may represent an opportunity lost for earlier acute oncology or MUO/CUP team involvement to help identify patients with poor prognosis or who are untreatable, rationalise investigations and involve CNS support and palliative care at the earliest opportunity. Once referred to the MUO/CUP team, investigations were arranged as outpatients where possible and biopsy, MDT review and oncology clinic were shown to be no slower than for those investigated as inpatients. Such findings highlight the need for the continuing development of acute oncology/MUO teams within all hospitals and ongoing education about cancer prognostic factors and the role of MUO/CUP teams amongst the wider clinical body.

Ninety-four per cent of patients presented with symptoms from their metastatic disease and 60% of referrals were made during inpatient admissions following emergency presentations, consistent with rates seen in previous national studies [1]. It is well known that outcomes for patients presenting acutely with a new cancer diagnosis are worse than in those detected by screening or outpatient ‘suspicion of cancer’ referral pathways [12, 14]. In our cohort, survival was poor across all patients diagnosed with presumed or confirmed malignant disease, but those referred during inpatient admissions demonstrated the worst outcomes. These findings strengthen the need for early specialist and CNS involvement and coordinated, streamlined and realistic diagnostic pathways in this patient group.

These data also highlight the urgent need for improved outpatient referral and diagnostic services to expedite investigations and diagnosis prior to clinical deterioration and emergency admission. Such ambitions have been the focus of several initiatives including the Accelerate, Coordinate, Evaluate programme in the UK aimed at improving the early diagnosis of cancer [15–17]. The emergence of rapid access diagnosis clinics (RADC) or multidisciplinary diagnostic centres (MDC) in some areas, including those specifically for patients with non-specific but concerning or ‘vague’ symptoms, may provide a model for patients who do not fit cancer-specific ‘suspicion of cancer’ referral criteria [16, 18, 19]. Notably, the cancer diagnosis rate frequently reported by RADC, MDC and 2-week-wait referral pathways, including patients referred with ‘vague symptoms’, is about 8% [16]. Our MUO/CUP oncology service uses ‘radiological suspicion of cancer’ as the key referral criteria, with a final presumed or confirmed cancer diagnosis rate of 92%. This underlines the importance of early access to diagnostic imaging for patients and highlights the need for collaborative working between diagnostic teams and oncology services for patients with advanced cancer, including direct referrals from GPs [20].

Significantly, in 8% of patients referred to the CUP service with radiological suspicion of cancer, a cancer diagnosis was not ultimately reached. As may be expected, more favourable survival was seen in this group, with 80% alive at 1 year. However, as a firm alternative diagnosis is not always reached, a non-cancer diagnosis may be one of exclusion and these patients often undergo sequential imaging and invasive procedures, with persisting uncertainty for patient and clinician.

Survival amongst patients in whom a primary cancer was identified was lower than may be expected. The majority of our patients presented with symptomatic metastatic disease, suggestive of late-stage advanced cancer compared to asymptomatic metastases detected during routine cancer staging. In addition, there were higher rates of emergency presentations in our cohort (54%) than that seen in unselected cancer patients (20%). Median survival of those presenting as emergencies was only 4.8 months, with 1-year survival lower than that previously reported in such patients (26 vs 38%). These differences may reflect more aggressive tumour biology or clinical behaviour manifesting as atypical presentations and leading to an initial diagnosis of MUO. Of those with a confirmed primary site, those with non-epithelial cancers had improved survival compared to epithelial cancers, confirming the importance of good pathological evaluation of biopsies, including morphology and IHC, within an MDT setting. Notably, only 53% of patients with a confirmed primary cancer were treated with SACT, despite referral to site specialist teams, likely reflecting the advanced stage of cancers presenting in this way. Similarly, patients with CUP may present initially to other cancer MDTs and undergo investigations relevant to that cancer site before referral. Ten per cent (*n* = 122) of all referrals in our cohort came from other cancer MDTs, and approximately half (*n* = 57) of these were confirmed to have CUP following comprehensive MUO/CUP team review. As the prognosis in this group of patients is so guarded, there is a clear need for expedited referral processes between specialist cancer MDTs and MUO/CUP teams, in both directions, to streamline investigations and avoid delayed specialist oncology review.

For the subgroup of patients with cCUP in our cohort, about a quarter was classed as being in the ‘favourable prognosis’ subgroup [3]. This is higher than previously reported, but includes, amongst others, the recently identified favourable colorectal phenotype not represented in previous studies [9, 10, 21]. However, in our experience, due to greater MDT working and oncology site specialisation, many patients found to have ‘favourable prognosis’ CUP are referred directly to site specialist teams, without prior referral or discussion in the CUP MDT. The main ‘favourable prognosis’ patients referred to the ECC CUP team were those with colorectal phenotype adenocarcinoma (mostly with advanced peritoneal metastases). This likely reflects the dual tumour-site specialisation of our CUP consultant oncologists.

Median survival for all cCUP patients was 4.0 months and only 10.9 months in those treated with SACT, which was significantly shorter than that previously reported for these patients [8–13]. This most likely reflects the highly inclusive ‘real-world’ referral criteria for our CUP service, including frail patients with advanced cancer who would not have been suitable for trials on which previous survival data are based. SACT can take many weeks to take effect, rendering it futile if predicted prognosis is short, and an active ‘best supportive care’ approach is preferred. Surprisingly, only 29% of patients with cCUP received SACT (24% with poor prognosis CUP and 43% with favourable prognosis CUP), with comparable outcomes across both groups. Assuming all patients were clinically suitable for comprehensive investigations at initial presentation, it is possible that the delay between first presentation and oncology review remains too long, with the window of opportunity for treatment missed. There can still be an undue focus on ‘finding a primary’, which can result in over-investigation, referral to multiple MDTs and loss of ‘ownership’ of the patient, sometimes with protracted diagnostic pathways. It can delay realistic discussions and patient-centred management, including timely involvement of palliative care. When a patient is fit for treatment, a prompt biopsy of the most accessible site, and detailed pathological evaluation, is the primary investigation required for SACT discussions. Further targeted investigations can be performed later if appropriate, and systemic treatment tailored accordingly. When a patient is not fit for treatment, the pathological confirmation of cancer is still important for some, but for others radiological diagnosis is adequate. Careful assessment of patients’ PS and prognostic markers requires constant re-evaluation when embarking on new investigations. CUP team cancer nurse specialists can help streamline diagnostic pathways, support patients through uncertainty and enable honest conversations in a patient-focused way. Improved understanding of prognosis from real-world datasets and validated prognostic biomarkers may aid such discussions and help avoid the over-investigation of unfit patients or those with poor prognosis [22]. Targeted investigations, efficient pathways and early discussion by a flexible and engaged CUP MDT can enable a rapid ‘best-fit’ diagnosis and early access to treatment discussions for fitter patients.

The present study does have limitations. The ECC CUP service is well established with strong links to primary and secondary care services throughout the South East Scotland Cancer Network. However, patients with MUO/CUP may present to many different services and many referring physicians feel able to assess a patient’s suitability for comprehensive investigation and have holistic discussions to this effect with patient and family, without oncology input. These cases may not come to the attention of the CUP team. This is compounded by ongoing confusion regarding diagnostic labels, variability in ICD coding of MUO/CUP and subsequent recording of patients in local or national cancer registries. We also recognise the diversity of MUO/CUP services across the UK, including significant differences in referral criteria. Whilst the data presented here may not be representative of the patient populations encountered by specialist tertiary referral CUP services, we feel it is relevant to primary and secondary care health professionals and acute oncology teams who first encounter patients with MUO. We believe that through the creation of CUP specialist teams, multidisciplinary input, CNS support and research interest, the NICE guideline, 2010 has improved the management of patients presenting with MUO/CUP. We strongly support work to expedite and streamline investigations for patients presenting with suspected cancer and to facilitate onward referrals to oncology teams. We would advocate future work to improve the collection of standardised clinical information on patients with MUO/CUP, in order to aid research and improve outcomes.

## Conclusion

The ECC CUP team was established in response to the release of NICE guidance for MUO/CUP in 2010. This 10-year review demonstrates the poor outcomes for patients presenting with new symptomatic advanced cancer of undefined primary origin and the continuing relevance of this national guidance. It highlights the need for early secondary care and oncology referral when imaging reports suspicion of metastatic cancer. It also confirms the benefits to primary and secondary care teams of having access to rapid diagnostic capability and dedicated multidisciplinary MUO/CUP oncology services. Patients need a patient-centred, streamlined experience with CNS support, prompt senior decision making and early involvement of palliative care teams. Improved understanding of outcomes in patients presenting with MUO, and the characterisation of patient diagnostic groups, helps inform shared discussions with our patients and colleagues about investigations and treatment.

### Reporting summary

Further information on experimental design is available in the Nature Research Reporting Summary linked to this paper.

## Supplementary information

Supplementary Data

Reporting summary checkllst

## Data Availability

The datasets used and/or analysed during the current study are available from the corresponding author on reasonable request
