# Supplementary material for: Characterisation and outcomes of patients referred to a regional cancer of unknown primary team: a 10-year analysis
Source: Br J Cancer. 2021 Sep 6;125(11):1503–10. doi: 10.1038/s41416-021-01544-1 (PMC8608886; doi:10.1038/s41416-021-01544-1)
Supplement: Supplementary file 1 — Supplementary Data [file 41416_2021_1544_MOESM1_ESM.pdf]

Supplementary Figure 1: Pathway and diagnostic subgroups of patients referred to the ECC Cancer of Unknown Primary Team over 10 years

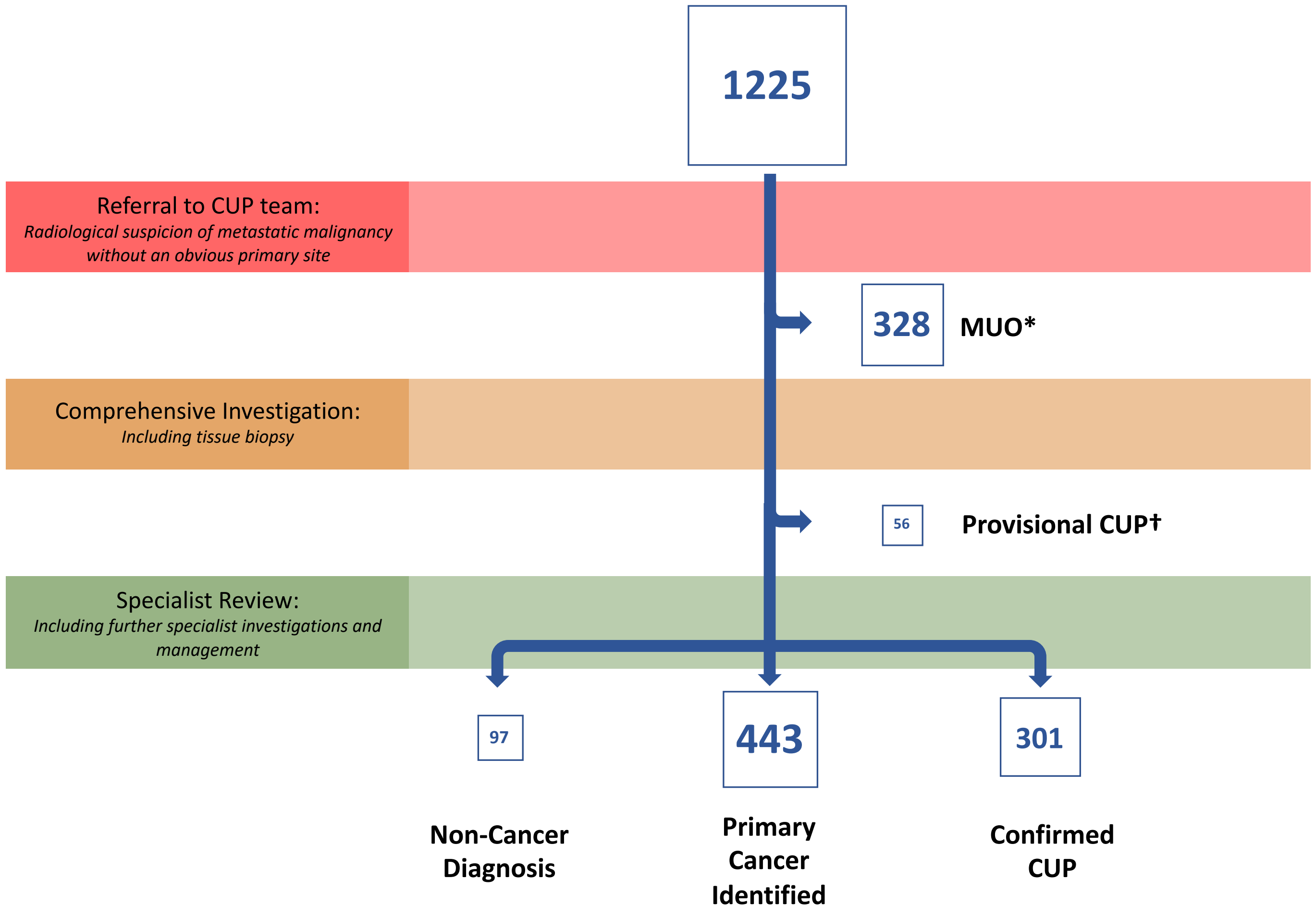

MUO* - comprehensive investigation, including biopsy, not clinically appropriate; pCUP † - comprehensive investigation, including biopsy, complete but patient not fit to be seen in specialist CUP clinic

Supplementary Figure 2: Kaplan Meier curves examining survival of patients referred to the ECC CUP team by route of referral

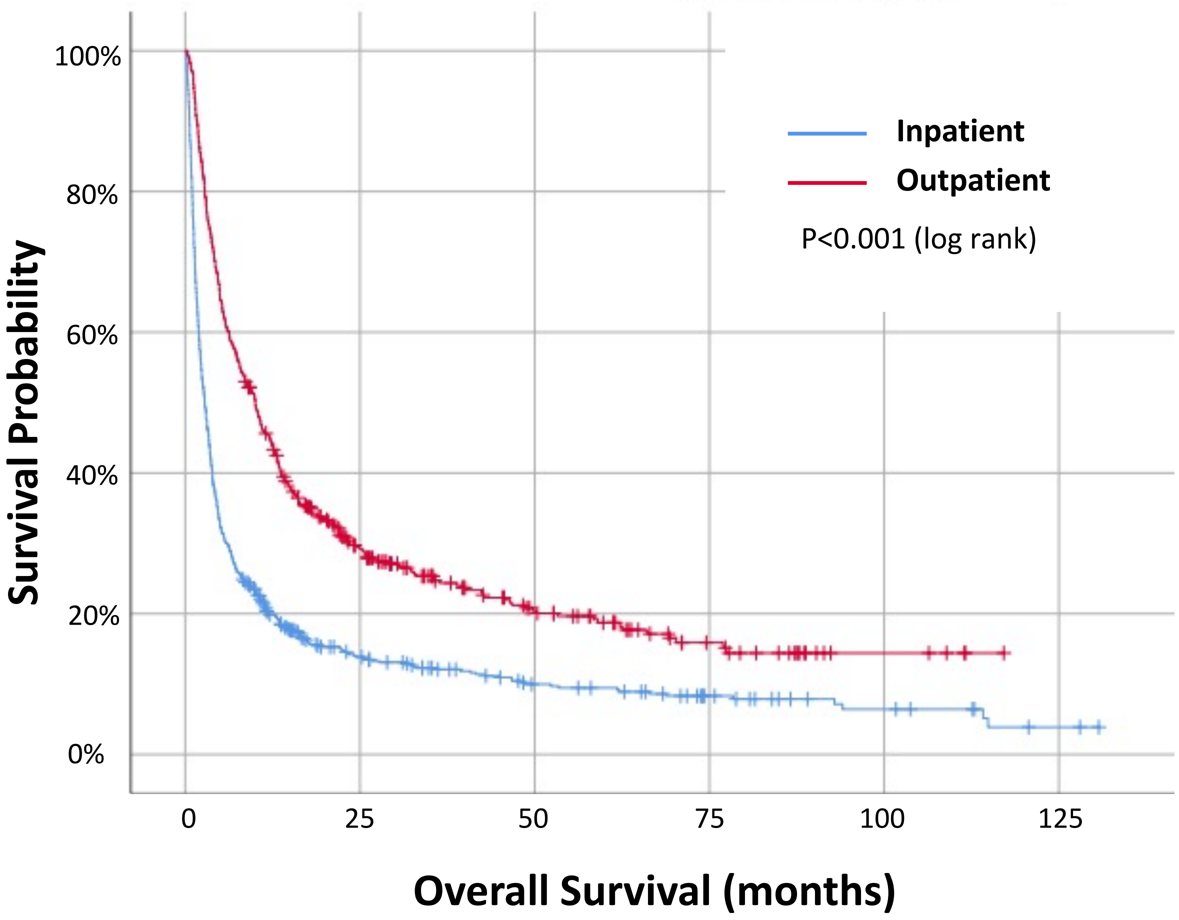

Supplementary Figure 3: Kaplan Meier curves examining survival for all diagnostic groups, sub-dividing Primary Cancer Found into epithelial and non-epithelial* malignancies

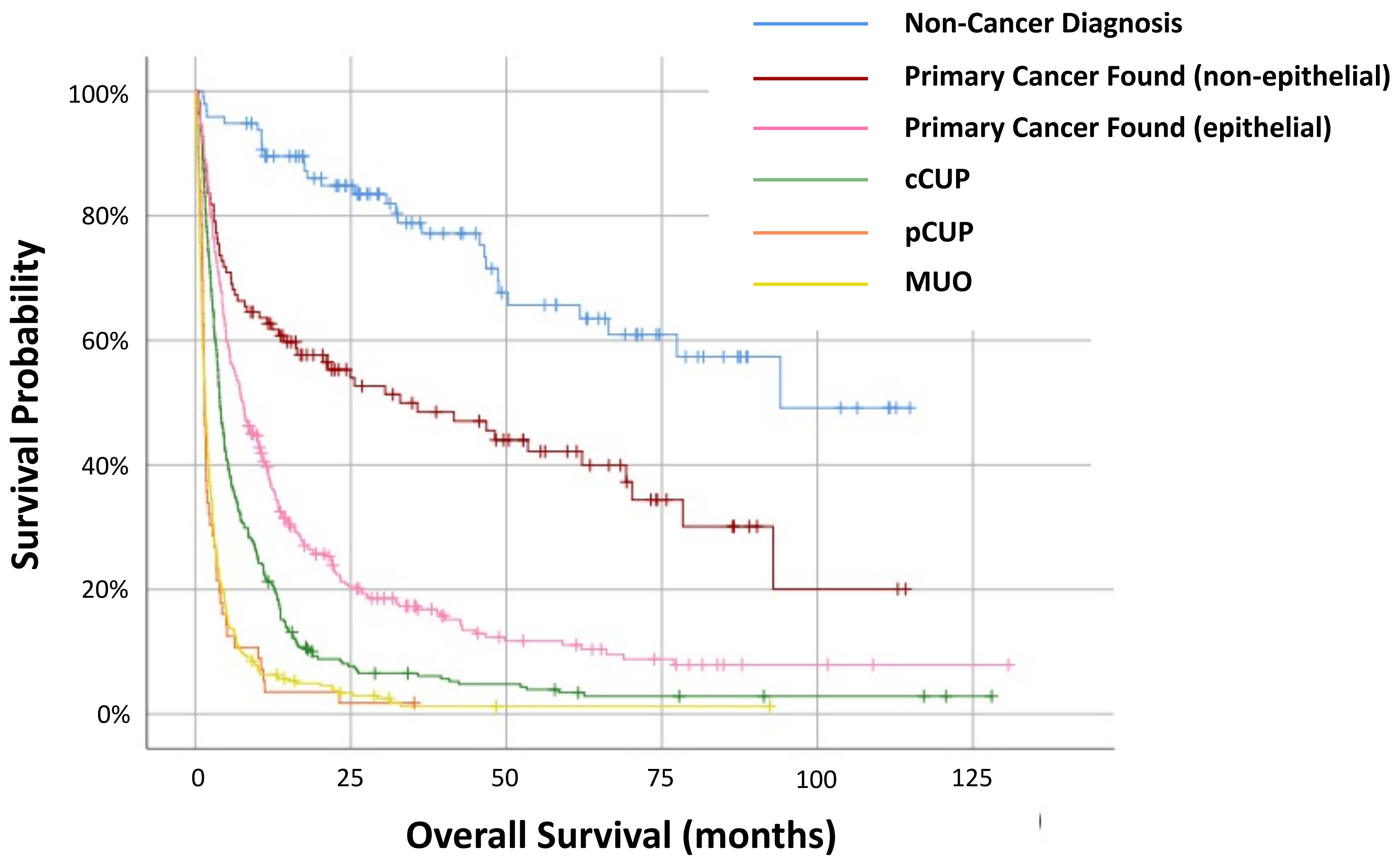

Non-epithelial malignancies * - lymphoma, myeloma, sarcoma, melanoma

Supplementary Figure 4: Kaplan Meier curves examining survival of patients with cCUP by clinicopathological prognostic group

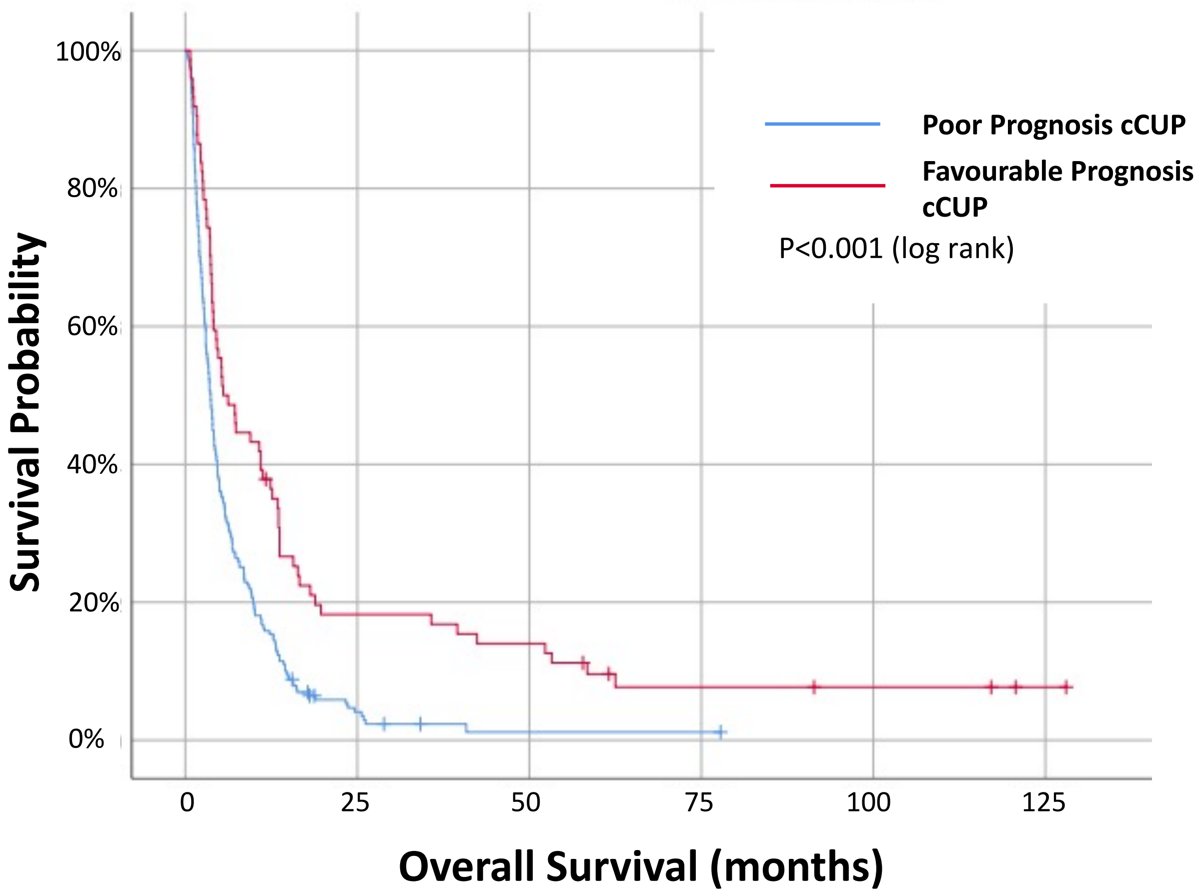

Supplementary Figure 5: Kaplan Meier curves examining survival of patients with cCUP treated with systemic anticancer therapy by clinicopathological prognostic group

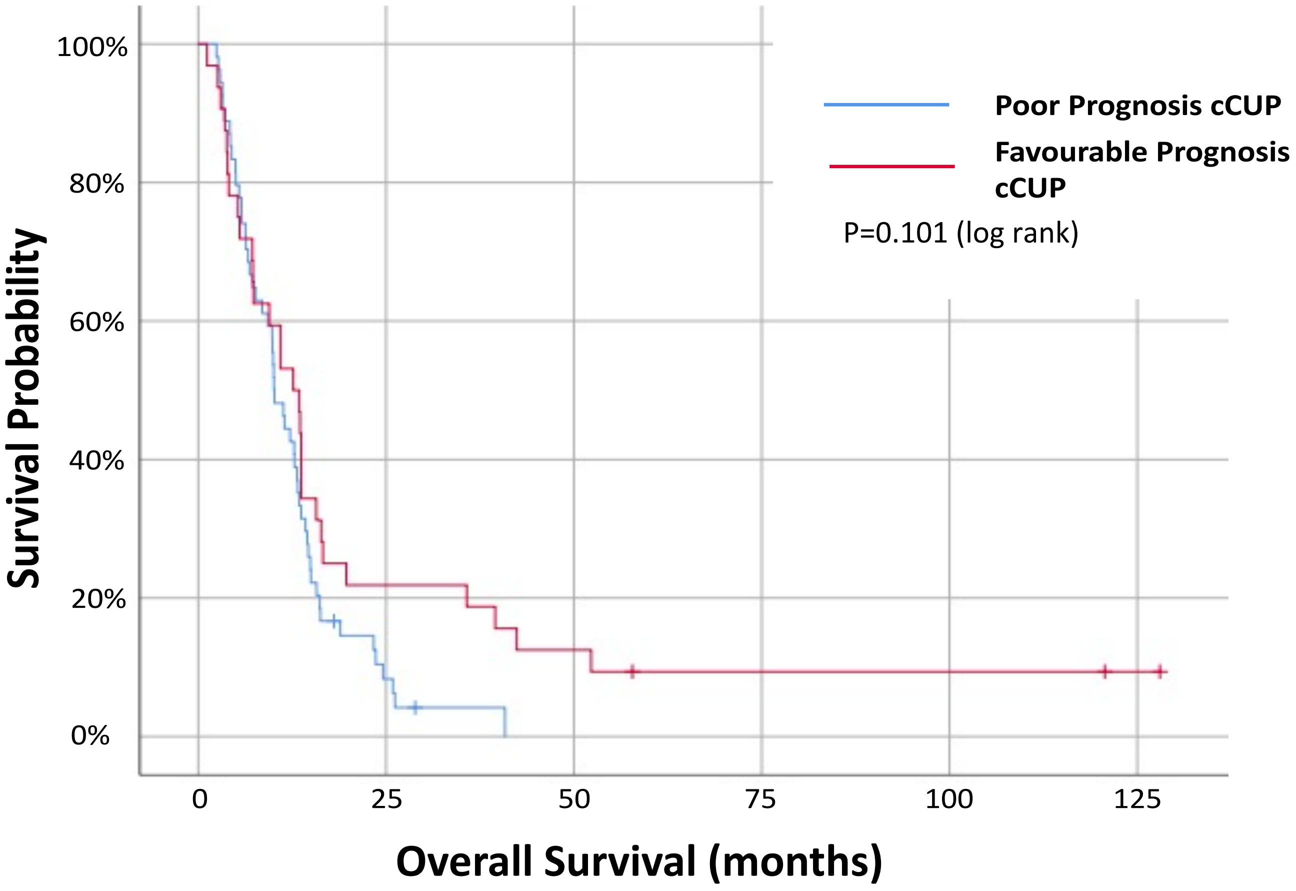

Supplementary Table 1: Reasons patients with a final diagnosis of MUO did not undergo comprehensive investigations and associated median survival

Reason for not undergoing comprehensive investigation	n (%)	Survival (Months) median (IQR)
**Frailty***	262 (80)	1.4 (0.7-2.9)
**Patient wishes**	39 (12)	2.3 (1.2-3.9)
**Biopsy attempt unsuccessful**	17 (5)	3.5 (1.6-6.4)
**No accessible lesion**	10 (3)	4.7 (2.9-10.4)

Frailty* was broadly defined as poor performance status and/or clinical unsuitability for invasive investigation or systemic anticancer therapy

Supplementary Table 2: Source of referrals to the ECC CUP by route of referral

			All	Non-Cancer Diagnosis	Primary Cancer Identified	Confirmed CUP	Provisional CUP	MUO
			**1225**	**97**	**443**	**301**	**56**	**328**
			**n (%)**	**n (%)**	**n (%)**	**n (%)**	**n (%)**	**n (%)**
**Source of Referral**	**Medicine**	**All**	**714 (58)**	**59 (61)**	**237 (53)**	**153 (51)**	**31 (55)**	**234 (71)**
		I/P	*555 (45)*	*37 (38)*	*177 (40)*	*106 (35)*	*27 (48)*	*208 (63)*
		O/P	*159 (13)*	*22 (23)*	*60 (13)*	*47 (16)*	*4 (7)*	*26 (8)*
	**Surgery**	**ALL**	**241 (20)**	**12 (12)**	**89 (20)**	**74 (25)**	**19 (34)**	**47 (14)**
		I/P	172 (14)	8 (8)	56 (13)	52 (17)	17 (30)	39 (12)
		O/P	67 (5)	4 (4)	31 (7)	22 (8)	2 (4)	8 (2)
	**General Practice**	**ALL**	**132 (11)**	**17 (18)**	**68 (16)**	**16 (5)**	**3 (5)**	**28 (9)**
		I/P	*n/a*	*n/a*	*n/a*	*n/a*	*n/a*	*n/a*
		O/P	*132 (11)*	*17 (18)*	*68 (16)*	*16 (5)*	*3 (5)*	*28 (9)*
	**Other Cancer MDT**	**ALL**	**122 (10)**	**7 (7)**	**41 (9)**	**57 (19)**	**3 (5)**	**14 (4)**
		I/P	*10 (1)*	*n/a*	*5 (1)*	*4 (1)*	*0 (0)*	*1 (0)*
		O/P	*112 (9)*	*7 (7)*	*36 (8)*	*53 (18)*	*3 (5)*	*13 (4)*
	**Radiology**	**ALL**	**16 (1)**	**2 (2)**	**8 (2)**	**1 (0)**	**0 (0)**	**5 (2)**
		I/P	*1 (0)*	*0 (0)*	*1 (0)*	*0 (0)*	*0 (0)*	*n/a*
		O/P	*15 (1)*	*2 (2)*	*7 (2)*	*1 (0)*	*0 (0)*	*5 (2)*

I/P – referred as inpatient; O/P – referred as outpatient

Supplementary Table 3: Presenting complaints prompting initial radiological investigations in patients referred to the ECC CUP team

		All	Non-Cancer Diagnosis	Primary Cancer Identified	Confirmed CUP	Provisional CUP	MUO
		**n (%)**	**n (%)**	**n (%)**	**n (%)**	**n (%)**	**n (%)**
**Selected Presenting Complaints**	**Pain**	650 (53)	41 (42)	247 (56)	182 (60)	32 (57)	148 (45)
	**Weight Loss**	509 (42)	23 (24)	162 (37)	127 (42)	26 (46)	171 (52)
	**Altered Bowel Habit**	151 (12)	4 (4)	51 (12)	46 (15)	10 (18)	40 (12)
	**Palpable Lump/Mass**	117 (10)	3 (3)	15 (11)	42 (14)	4 (7)	18 (5)
	**Hypercalcaemia**	51 (4)	2 (2)	14 (3)	14 (5)	3 (5)	18 (5)
	**Spinal Cord Compression**	55 (4)	1 (1)	27 (6)	17 (6)	0 (0)	10 (3)
	**Other Neurological Symptoms***	118 (10)	9 (9)	61 (14)	11 (4)	0 (0)	37 (11)
	**Pathological Fracture**	16 (1)	2 (2)	6 (1)	3 (1)	1 (2)	4 (1)

Other neurological symptoms* - included limb weakness, sensory disturbance, visual disturbance, seizures

Supplementary Table 4: Sites of disease on initial radiological investigations in patients referred to the ECC CUP team

		All	Non-Cancer Diagnosis	Primary Cancer Identified	Confirmed CUP	Provisional CUP	MUO
		n (%)	n (%)	n (%)	n (%)	n (%)	n (%)
All Patients	*Patients*	*1225*	*97*	*443*	*301*	*56*	*328*
	Adrenal	94 (8)	2 (2)	43 (10)	21 (7)	4 (7)	24 (7)
	Bone	360 (29)	36 (37)	148 (33)	78 (26)	10 (18)	88 (27)
	Brain	114 (9)	12 (12)	44 (10)	13 (4)	0 (0)	45 (14)
	Liver	501 (41)	16 (16)	147 (33)	124 (41)	22 (39)	192 (59)
	Lung	372 (30)	20 (21)	126 (28)	89 (30)	14 (25)	123 (38)
	Peritoneal	224 (18)	7 (7)	56 (13)	72 (24)	22 (39)	67 (20)
	Thoracic LN	206 (17)	5 (5)	72 (16)	75 (25)	9 (16)	45 (14)
	Cervical/Axillary LN	98 (8)	5 (5)	39 (9)	39 (13)	4 (7)	11 (3)
	Abdominal/Pelvic LN	343 (28)	10 (10)	133 (31)	101 (34)	13 (23)	86 (26)
Single Site of Disease	*Patients*	*469*	*70*	*169*	*100*	*26*	*104*
	Adrenal	3 (0)	0 (0)	3 (1)	0 (0)	0 (0)	0 (0)
	Bone	126 (27)	27 (39)	57 (33)	17 (17)	4 (16)	21 (20)
	Brain	48 (10)	6 (9)	15 (9)	5 (5)	0 (0)	22 (21)
	Liver	98 (21)	11 (16)	33 (20)	15 (15)	6 (24)	33 (32)
	Lung	20 (4)	8 (11)	3 (1)	2 (2)	1 (4)	6 (6)
	Peritoneal	63 (13)	4 (6)	13 (8)	27 (27)	7 (28)	12 (12)
	Thoracic LN	13 (3)	2 (3)	4 (2)	7 (7)	0 (0)	0 (0)
	Cervical/Axillary LN	18 (4)	0 (0)	10 (6)	5 (5)	2 (8)	1 (1)
	Abdominal/Pelvic LN	33 (7)	4 (6)	11 (7)	13 (13)	2 (8)	3 (3)

**Single Lesion**	***Patients***	*91*	*22*	*32*	*14*	*1*	*21*
	**Adrenal**	1 (2)	0 (0)	1 (3)	0 (0)	0 (0)	0 (0)
	**Bone**	33 (32)	13 (59)	9 (29)	4 (29)	0 (0)	7 (33)
	**Brain**	15 (16)	3 (14)	5 (16)	1 (7)	0 (0)	6 (29)
	**Liver**	10 (10)	3 (14)	4 (13)	0 (0)	1 (100)	2 (10)
	**Lung**	1 (1)	1 (5)	0 (0)	0 (0)	0 (0)	0 (0)
	**Peritoneal**	0 (0)	0 (0)	0 (0)	1 (7)	0 (0)	0 (0)
	**Thoracic LN**	1 (1)	0 (0)	0 (0)	1 (7)	0 (0)	0 (0)
	**Cervical/Axillary LN**	4 (4)	0 (0)	3 (9)	0 (0)	0 (0)	1 (5)
	**Abdominal/Pelvic LN**	2 (2)	0 (0)	1 (3)	1 (7)	0 (0)	0 (0)

Includes sites seen in >5% of all patients.

LN – lymph nodes.
